# Why some donors are more willing to donate platelets? ----a qualitative study on 25 regular platelet donors in Guangzhou, China

**DOI:** 10.1186/s12889-019-7783-0

**Published:** 2019-12-12

**Authors:** Chengpu Yu, Joseph Tak-fai Lau, Weixing Zhong, Xinting Huang, Chao Pan, Yu Chen, Huanpeng Lu, Fengxiao Zhao, Siying Zeng, Jinglin Lai, Chengwei Tie, Xu Li, Jianlin Zhang, Chuangqi Zhang, Ying Liu, Qianli Jiang

**Affiliations:** 1 The Department of Anthropology, Sun Yat-sen University, Guangzhou, China; 2 The School of Public Health & Primary Care, The Chinese University of Hong Kong, Shatin, Hong Kong, China; 3 The Southern Medical University, Guangzhou, China; 4 The Southern Medical University Nanfang Hospital, Guangzhou, China; 5 The Institution for Hospital Management of Tsinghua University, Beijing, China

**Keywords:** Platelet donation, Whole blood donation, Mutual assistance blood donation, China

## Abstract

**Background:**

Since 1998, China has gradually moved toward voluntary uncompensated blood donation. In some cities, the shortage of platelets has been noticeably severe. Mutual assistance that collects blood from one’s family and social networks is a potential solution. The measure, however, turned out problematic. There are donors who choose to donate platelets over whole blood without compensations, and donate platelets directly to blood banks instead of via the mutual assistance system. This study explores reasons behind their choices qualitatively.

**Methods:**

This report is based on data conducted from January to February 2018; 25 uncompensated regular platelet donors were interviewed. The blood component donation service team in Guangzhou facilitated the data collection process and referred prospectively eligible blood donors to our research team. The interviews took about 30 min to two hours to complete. The qualitative data were analyzed by using the software ATLAS.ti 8.

**Results:**

Platelet donation takes a much long time than whole blood donation and requires complicated processes. It may also cause discomfort as the other blood components are returned to the body, causing physical and psychological distress due to worries about contamination. Thus, platelet donation tends to involve higher time and psychological costs than whole blood donation. Yet, it has short collection intervals that allows for more frequent donations, and urgency of a severer shortage than whole blood. Hence, regular platelet donors may feel higher significance in platelet donation than whole blood donation, with the belief that more lives would be saved. Some whole blood donors thus switched to become platelet donors. Mutual assistance blood donation was not chosen by the participants for platelet donation, because such donations may exert moral pressure to both the donors and recipients. Furthermore, “acquaintance” has been loosely defined; the system has sometimes been manipulated to become profit-making monetary transactions. It hence failed.

**Conclusions:**

The practice of platelet donation reinforces the understanding that blood donation is a gift giving process performed among strangers. A safe and sustainable voluntary blood supply can only be secured in the absence of monetary transactions and moral pressure.

## Background

Since the implementation of the *Blood Donation Law of the People’s Republic of China* in 1998, China has gradually moved toward voluntary uncompensated blood donation (VUBD) [1]. There were about 14.6 million blood donors in China in 2017. The nation’s blood donation rate was 11‰ (per thousand), which was slightly higher than the 10‰ proposed by the World Health Organization(WHO) [2], but far below that of some high (32.6‰) and middle (15.1‰) income countries [3]. There is a strong demand for platelet donation as patients with various diseases (e.g. leukemia, aplastic anemia) require continuous infusion of platelets to maintain their life, and there is a shortage of supply in a number of major cities in China. The shortage can be attributed to multiple difficulties in collecting platelets through blood donation, such as the short preservation period (5 days), the long physical examination and acquisition time required (about two hours), stricter donation criteria, and higher collection and processing costs (about 1000 RMB or 140 USD for one episode). The lack of knowledge and misconceptions may also cause unwillingness to choose donating platelets over whole blood.

Mutual assistance blood donation (MABD) and general VUBD are two key means of collecting platelets from the general public. For instance, VUBD made up 60% of the supply in 2016 in one city of China [4]. MABD, supported by the country’s Blood Donation Law (article number 15;1998), intends to collect blood from people’s network members (e.g. family members, friends and co-workers), who would serve as primary donors during emergencies. MABD was used as a major source to collect platelets in some cities in China [5]. However, due to various difficulties in implementation such as transformation into profit-making monetary blood transactions, the scheme was recently abolished in most of the Chinese cities by the Chinese government on March 31, 2018. However, WHO data revealed that some developing countries still relied heavily on family blood donors during 2008 to 2015 [3]. In literature, there is a dearth of studies that looked at platelet recruitment strategies and reasons for platelet donation [6–9]. To our knowledge, the socio-cultural obstacles against collecting platelets through MABD have not been studied qualitatively. This study investigated why regular platelet donors preferred to donate platelets over whole blood and reasons for practicing VUBD for platelet donation over MABD.

## Methods

Twenty-five regular platelet donors were in-depth interviewed during January to February 2018. A semi-structured interview guide including the participant’s demographic information and blood donation experience was used (Additional file 1). Inclusion criteria were: 1) > 3 times of platelet donation through VUBD and at least once in the last 12 months, and 2) pledged at least one platelet donation within the next year. Interviews and data analysis were conducted by the first author (an anthropologist) and the corresponding author (a hematologist); recruitment was conducted by 13 medical students of the Guangzhou Southern Medical University. The blood component donation service team of the Guangzhou Blood Donor Volunteer Service Team connected us to eligible participants. By appointment and at convenient venues, the interviews were conducted (30 min to 2 h) in private settings. Two research staff assisted the key interviewer to record observations and asked supplementary questions. The interviewers briefed the participants about the purpose and logistics of the research and answered participants’ questions. Every participant received a small gift as a symbolic incentive. With written informed consent obtained from the participants, we tape-recorded all conversations. Ethical approval was obtained from the Department of Anthropology of the Sun Yat-sen University(NO:L20180103).

The audio-recorded interview data were transcribed verbatim by the 13 medical students to capture cultural concepts and nuances embedded in the language by coders who had familiarized themselves with the raw information and developed a draft code list independently to check consistency. The first and the second author who received qualitative and ethnographic training then reviewed the transcripts to ensure accuracy. To keep the data grounded and develop content sensitivity, new codes were added and unfit codes were discarded in a continuous manner. They compared their code list to resolve any discrepancies; the final main codes were then determined according to the agreed common themes. All the above steps were undertaken with the software ATLAS.ti 8 used for qualitative data processing and analysis. The key findings were translated into English by an independent professional bilingual translator who was not a member of the research team; another independent bilingual person back-translated the English version into Chinese. These two commercial translators were paid. The versions were then compared and revised by the second author.

## Results

### Switching from whole blood donation to platelet donation

Of the 25 participants, with one exception, all began blood donation with whole blood donation. When they were informed (mainly by the nurses of blood bank) that blood donation includes blood component donation and whole blood donation, they started donating platelets.

#### Platelet donation is also donation of time

The 25 participants included thirteen ordinary workers, two students, two medical workers, two Internet-based businessmen, one retired person, one civil servant, and four with unknown occupations. They mentioned that they had plenty free time. Time is an important consideration as the entire platelet donation process takes two or more hours to complete. It requires special equipment and venues, and detailed physical examinations for the volunteers to ensure compliance with the requirements as the collection cost is high (about 1000 RMB or 140 USD per episode). The process of plateletpheresis takes another 45 min to complete. Beyond any doubt, platelet donation is much more time consuming than whole blood donation. Availability of free time is likely to affect decisions for platelet donation.

The case of Respondent 1 (female) is illustrative of the importance of time. She was a retired soldier and then changed her job to work at the Food and Drug Administration of Guangzhou. She had donated whole blood for 24 times, amounting to 7600 ml. She knew about platelet donation, but due to time constraints, she had never donated platelets until July 2012, when she had more free time upon returning from a 3-month sea voyage. On 25th of July, 2012, she donated platelets for the first time and till the interview, five times. She said:

“*Originally I always felt that it took a long time to donate platelets, and I had to take time to work it out. I had previously worked in the army. Time was relatively tight, so I had never donated platelets. I felt that the time for donating too long, so I did not do it. After I changed my job, I began to donate platelets*.” (Respondent 1, 20180203)

Respondent 2 (female) began donating whole blood since 2000. After many times of donating whole blood, she tried to donate platelets. She concluded that unlike whole blood donation, donating platelets takes a lot of time.

“*I think at least individuals need to reserve time in advance. It’s unlike the whole blood which can be done in a few minutes. Donating platelets takes about one or two hours, during that period, the donor has to stay there and wait, which is a certain time cost. That is the main difference.*” (Respondent 2, 20180202)

Respondent 3 (female), a 30-year-old single mother, began to donate whole blood since 2007 and turned to blood component donation in December 2009. After she gave birth to her child, she did not donate blood for more than 2 years because she had to take care of him. Currently, the boy was about 6 years old; she sold agricultural and sideline products online, so she was quite free. She went to donate platelets mostly with the boy, as there was no available caretaker and she wanted the boy to know about the process of donating blood since childhood so that he would donate blood after 18 years old. She had donated platelets for 59 times. (Respondent 3, 20,180,128).

These cases show that regular platelet donors usually have less demanding time schedules. To other people, time is hence a critical potential barrier against platelet donation.

#### Auto blood re-transfusion, anxiety and discomfort

The extracted blood enters a strictly disinfected equipment that separates platelets from other blood components; the latter is re-transfused back to the donor’s body. This additional returning process may cause physical discomforts to the donor, such as nausea, palpitation, and dizziness. Furthermore, anti-coagulants are added to the blood to prevent clotting. As the anti-coagulant binds to calcium ions, the decrease in blood calcium may cause tingling lips or make the donors feel cold. In addition, donors may worry about hygienic conditions and infections, as the blood has left the body and might have been contaminated by micro-organisms such as HIV and HBV. For instance, the window period for detection of virus such as HIV is a potential threat.

Respondent 1 mentioned that when she first donated platelets, the biggest concern was safety. She said: “*Before you know about platelets, you feel more eager about it, thinking that it goes through the machine, worrying that my blood is safe or not. Undoubtedly we may have some concerns. Before blood donation, I imagined that the blood be drawn from one arm and returned from the other arm, that made me worry about the safety of the tube. I knew that it was a one-off thing, but I felt like it was unsafe. At the time, I was worried that although all the diseases were detected, there was still a period of time when they could not be detected, for example the window period. I thought there was a certain risk. It was also a small worry for me. But now I don’t have this worry at all. I guess other people may have the similar feel as mine.*” (Respondent 1, 20,180,203).

Respondent 4 (female) is a university student. Unlike other donors, she had never donated whole blood before she first donated platelets. She not only was concerned about safety, but also experienced a serious physical reaction. “*Even if you have understood these things well, there are still some concerns. After all, there is a cyclical process for platelet donation. Although medical care is good enough, the equipment safety is guaranteed, most people are worrying that it’s difficult to guarantee one hundred percent safety, and the same to me. What’s more, the first time when I donated blood, I donated platelets instead of whole blood, which brought me even more serious reaction. I was feeling cold and a little shivering at the time. They said that I was the one who reacted most seriously.*”(Respondent 4, 20,180,129).

Respondent 5 (male) first donated whole blood on his 20th birthday. After the introduction by nurses, he knew about platelets. One day in 2000, the blood bank called him and asked if he was willing to donate platelets. He was very scared and even called a classmate to accompany him. He said: “*You can take out my blood, but it is scared when you return it to my body after an unknown process. I never know what’s going to happen? What has changed? Will there be any disease? What’s the side effect? etc. I was a bit worried about this. As my starting point was to save lives, so later I went and found that there was nothing.*” (Respondent 5, 20,180,201).

Respondent 6 (male) was working in administrative management in a company. He had hesitated again and again when he first donated blood. He felt that there was too much negative information on blood donation and his mother did not support it, saying that “a drop of blood, ten chickens”; blood is invaluable and cannot be lost. But he insisted that as a Communist, he should help others. After this experience, he felt that blood donation had caused no harm to the body and he persisted. However, he considered platelet donation be more “horrific” than whole blood donation.

“*In fact, for the first time, donating blood platelets was even more horrific than donating whole blood. To tell the truth, the first time I saw the machine, I was afraid. Because the blood of red color goes in, it becomes yellow, and it will reenter my body. This was terrible. At the beginning, our volunteer friends encouraged me to donate platelets. I did not know what platelets were. I later learned that platelets were a component of the blood used to stop bleeding. But when I went to donate, I was really scared. I saw all kinds of pipes and yellow blood. The first time I donated only one treatment unit. I found that there was nothing wrong with my body and I insisted on donating platelets.*”(Respondent 6, 20180204)

The above-mentioned cases reflect that, even for regular whole blood donors, they still needed to overcome distress during their first donation of platelets, and they might need to deal with physical reactions. Unlike simple and fast whole blood donation, apheresis of platelets requires separation of blood components and re-transfusion; the complexity raises safety concerns. The change in blood color could also cause distress. Respondent 4 donated platelets without any experience of donating whole blood, the first-time donation caused even stronger fear and more serious physical reactions. Thus, the blood bank is more keen to encourage regular whole blood donors to become platelet donors, rather than encouraging inexperienced people to start blood donation with platelet donation.

#### Donation intervals, shortage, and significance

Why some whole blood donors turn to platelet donation? Platelet donors may believe that the deed has higher significance as there is a greater shortage and they can donate platelets more frequently than whole blood. The donor’s body recovers faster in platelet donation than whole blood donation; the donation interval is 6 months for whole blood donation but only 2 weeks for platelet donation. The shorter interval period is one of the greatest motivations to donate platelets and is also a common reason for switching to frequent platelet donation. It implies that platelet donors can potentially save more lives through more frequent blood donations, thus making the donations more meaningful. As Respondent 1 said:

“*Platelet means more. Because I can donate once more in half a month, then I may be... For this kind of blood donation, it is equal to saving more lives*.”(Respondent 1, 20180203)

Respondent 7 (male) was 58 years old. He began to donate blood regularly since 1979 in Hong Kong when he tried to demonstrate courage to his girl-friend in spite of fear for needles. He had donated whole blood and platelets for 168 times. He switched to platelet donation when he knew about the 2-week donation interval, and had donated platelets for 20 times in the last year. After donating platelets, he never again donated whole blood. He smiled and told us:

“*I don’t want to go back and donate whole blood. The interval time of whole blood is too long, which takes half a year. By donating platelets, we can make it every half a month. It is much faster and makes us happier.*”(Respondent 7, 20180201)

The shortage of platelets was another reason for some blood donors’ switching to platelet donation. Respondent 8 (female) worked in the transfusion department of a hospital and was knowledgeable about the medical procedures for blood collection and blood use. In the past, she had only donated whole blood for two times. Her first donation was conducted in a shopping mall when she saw a lot of people donating blood, and she joined them. When she donated whole blood for the second time, a nurse told her that there was a shortage of platelets and she could try to donate platelets later. Since 2006 and accordingly, she began to donate platelets, firstly in once a quarter, once a month, and then once every 2 weeks. She had become a regular donor and had donated platelets for more than 70 times. (Respondent 8, 20,180,204)

### Obstacles for MABD

#### “Moral debts” (*Renqing*,人情) and distress

MABD becomes important when blood banks have a shortage of platelet supply. The practice may result in moral or practical burdens to prospective and actual donors. For instance, Respondent 9 (female)‘s grand uncle had just undergone a surgery and needed infusion of platelets to stop bleeding. Her mother told her about this and she immediately rushed to the hospital and filled out a blood donation form for platelet donation. The two families were in a good relationship; the grand uncle who was a cadre supported her mother’s education and living during her poor childhood. Respondent 9, however, took some cold medicine a week ago and became non-eligible for platelet donation. When she visited her grand uncle in the hospital, she apologized: “I am terribly sorry as I failed to help you”. (Respondent 9, 20,180,206) She felt a moral obligation to donate platelets and experienced distress when she was unable to do so.

Respondent 10 (male), an ordinary employee, had donated whole blood for more than 10 time and platelets for 39 times since 2009. He had also participated twice in MABD; one episode was unsuccessful:

“*One time was that my friend’s mother was ill in the hospital and needed MABD. They found me at the time, but as I was just within a platelet-donation interval period, I couldn’t offer it. But we are old friends. I found a person in the service team to help him. The other time was that I saw the information on blood needs in the newspaper. It was like a leukemia patient or thalassemia patient. I helped that patient (a child) with MABD. His family wanted to give me compensation (money). I didn’t accept it and said that they could use this money to buy some eatables for the child, and wished him a quick recovery. Later we exchanged our phone numbers. They sent me messages of thanks and blessings during the holidays, but I didn’t usually reply, only in New Year’s time, I wished them happy new year and good health. From my point of view, I would not say that I had saved that child, nor I would care about what the child is doing now, because I am unable to care about his future development. I just passed by and gave him a hand*”. (Respondent 10, 20180202)

In the first event, Respondent 9 did not hesitate to agree to donate blood platelets to repay *Renqing*. She felt apologetic when she could not help. This *Renqing* pressure was also revealed in his episode of actual MABD in another manner. When Respondent 10 offered platelets to the child, the child’s family tried to send him money to express gratitude. After the child recovered, being morally motivated, the parents sent out greetings during festivals. However, from Respondent 10’s point of view, he was not willing to flaunt his behavior to save the child, and he had no ability to pay attention to his future development. The donor felt uncomfortable about the recipient’s expression of gratitude via tangible means and involvement in the child’s development.

MABD is usually conducted among acquaintance. Donors would be happy with successful outcomes, but might face distress if the medical procedure failed. Respondent 11 (male) worked in a lighting factory. He started donating whole blood in 1999 and turned to blood component donation in 2000. At the time of the interview, he had donated blood for more than 50 times. He had experienced MABD twice; both experiences were unpleasant to him. The first episode was a donation to an acquaintance’s son; he was given a large amount of money that he refused to accept and felt embarrassed. The second MABD was given to his cousin, who unfortunately did not survive. Given these two unpleasant events, he said.

“*I hate MABD because the donor knows who he is helping and if the person he wants to help dies, he may feel uncomfortable. I have always insisted on donating blood to the blood bank. I don’t like this kind of one-on-one MABD. In such one-on-one situations, it is sad as it means that people around you have accidents. If I can’t help ultimately, I would feel very uncomfortable*. ” (Respondent 11, 20180201)

MABD is not given to strangers, instead to one’s acquaintances. The patient, the patient’s family, relatives, friends, and other people are all emotionally involved, creating potential moral debts and distress before, during, and after MABD.

#### Monetary transaction practices of MABD

As we mentioned above, MABD intends to collect platelets from the patient’s network members, who would serve as primary donors during emergencies. After they fill in the Mutual Blood Donation Registration Form and go to blood bank to donate platelets, the hospital will provide the same amount of platelets to the patient. However, MABD encounters at least the following obstacles in practice. First, mobilization is difficult as some out-of-town patients would be unable to ask their relatives and friends to donate blood in medical settings and high costs may be involved (e.g. transportation and *Renqin*g). Second, it is usually impossible to know whether the mobilized relatives and friends are suitable for the platelet donation. These difficulties cause some patients to buy platelets from brokers when they find it hard to recruit donors among their networks, or when they are unwilling to make such requests. The brokers arrange blood sellers to pretend to be the patient’ relatives to donate platelets through MABD; they charge the patient high prices (from hundreds to thousands RMB) but pay little money to the blood sellers. Thus, MABD has often been twisted into monetary transactions. In cases of severe medical incidences and in the absence of available platelet donation, patients’ families may feel morally obliged to seek blood brokers’ “help”. Respondent 12 (female), who had donated blood for many times and had participated in MABD showed understanding and sympathy about the helplessness and hardships of the patient’s family members who deal with blood brokers:

“*Nobody wants to spend extra money to buy platelets, but if you don’t buy them, you will have to look at your loved ones lacking blood supply. I know that blood brokers sell blood but I do not have the right to condemn them. Those family members do not have to thank us volunteers for blood donations as the blood has not been allocated to them. They will be grateful to the blood brokers and spend money to get the blood*.”(Respondent 12, 20180128)

As it is difficult to validate the relationships between donors and recipients in MABD, blood sellers can easily pretend to be the recipients’ friends or relatives under brokers’ arrangements. Faced with the severe shortage of platelets and obstacles, MABD has often deviated from its original intention of serving as a source of emergent supply to become monetary transactions.

#### Positive motivations for platelet donation

Despite the issues of mental distress, physical discomfort, and monetary transactions, some people have become regular platelet donors. According to the interviews, the motivation is backed up by the simple feeling that it saves lives and is a happy act. The following excerpts from the interviews clearly show this motivation.

“*Anyway, my blood can save people’s lives without sacrificing my own. So why not?*”(Respondent 5, 20180201)

“*In fact, it is very simple, to save people. I do not know who to save. In any case, I feel that blood donation can save people. So I insist on doing it, although sometimes I would not succeed, anyway I have tried my best.*”(Respondent 9, 20180206)

“*I think it can really help others. I donate blood and maybe save someone. People all say that’* *better save one life than build a seven-storey pagoda**’. Being nice is good for others as well as for oneself, especially when the patient recovers*.”(Respondent 11, 20180204)

“*At first, I think people are not just for their own sake in society, they can’t just get, and also need to make some contributions. I would think that although I have a bit of pain, I can help others, and it may not only help others, but it may also save a life. This is already worth it to me.*”(Respondent 12, 20180204)

“*Because it is a matter of health, so I donate blood. Every time after I donate blood, I have a happy mood and it prompts me to insist on blood donation. It is a virtuous cycle. It may be that helping others makes me feel good.*” (Respondent 1, 20180203)

“*The greatest impetus is that Guangzhou blood center needs more blood, patients need blood, and blood donation can save people.*” (Respondent 3, 20180128)

The difference between VUBD and MABD is that the former is a pure gift relationship between strangers, while the latter is potentially loaded with *Renqing*, moral pressure, and in some cases, monetary transactions. VUBD reflects the social value of donors, which is the positive motivation for them to donate platelets.

## Discussion

The findings suggest that two issues may need further discussion and analysis. First, why the donors would feel stressful when their blood components were re-transfused back to their bodies? This may stem from the fact that after the blood leaves the body, people would think that it may become unclean or even dangerous. The famous anthropologist M. Douglas has made a profound interpretation of filth and danger in her book *Purity and Danger*. She believes that people’s perceptions of filth include views on orderliness and disorder. It’s not because it is inherently unclean, but because it is contrary to the classification framework and social order that people follow [10]. For example, we do not think that our excretion is unclear until it leaves our body and is seen to be dirty. The same might be true of blood. We may worry about contamination. In apheresis platelets, the blood components flow through the vascular tract, being separated by the machine and returned to the body. The blood circulation outside the body is clearly in an improper place outside our body system. This is a plausible psychological and cultural root cause of people’s fear that it may be contaminated.

Second, the regular platelet donors were unwilling to participate in MABD as it may induce a moral burden, and it may be associated with monetary transactions. People may feel morally obligated to help friends and relatives who need blood transfusions by buying blood from brokers at high fees. Since there are no electronic linkages among blood collection systems of different cities, some blood sellers may donate blood in different cities under MABD without adhering to the recommended intervals, thus compromising blood quality and their health. Some blood donors might conceal their health status and introduce risks of transmitting infectious diseases to the recipients in the presence of window periods for detection [11].

Since the implementation of the Blood Donation Law in 1998, Chinese people have gradually established and accepted the concept of VUBD. As R. M. Titmuss puts it, in the presence of both blood donations and a blood market in a society, people’s common positive perceptions and values toward blood donation would dissociate. The transformation of MABD into monetary transactions would tarnish the concept of VUBD, diminish people’s wish to contribute to social good, and break the positive value orientation of existing blood donors [12].

## Conclusions

Some perspectives explained why regular platelet donors tend to perform VUBD over MABD, and to donate platelets instead of whole blood. Although platelet donation is more time-consuming and may cause discomfort, it has the advantages of short donation intervals and significance of severe shortage, hence the potential to save more people’s lives. MABD not only involves moral burdens, but may also have been turned into monetary transactions, which is against the wish of some regular blood donors.

Human blood is rare and precious. So far, we are unable to use artificial blood on a large scale for clinical blood transfusions. The majority of blood for clinical use is obtained from other people. VUBD, as a gift of life to a stranger, is regarded as perhaps the “purest model” of altruistic behavior [13]. Blood is seen by people as the source of life, which is not only for oneself but also for all human beings. Donating blood to save others’ lives has become a social good. At the same time, we also see that this act is appreciated by the entire society and various cultures; it brings spiritual joy to the blood donors [14, 15], who also donate blood to show that they are healthy. After they donate blood, they save lives and are happy. Therefore, blood donation is an act of benefiting others as well as oneself. The practice of platelet donation tells us again that blood donation is gift giving among strangers. Safe and sustainable blood supply can be guaranteed only through VUBD.

## Limitations

We acknowledge that the results of this study may not be generalized to the national scenario of Chinese platelet donation, given the study was conducted only in one city and its qualitative nature. Despite such limitations, we believe that this study has provided insightful and culturally informed data on whole blood donation and platelet donation, as well as VUBD and MABD. The observations remind blood banks to target prospective platelet donors who have more free time (e.g. college students and freelancers), and consider how to shorten the required time (e.g. appointments). Blood banks also need to strengthen safety and psychological counseling to reduce people’s physical and psychological negative reactions to platelet donation.

## Supplementary information

**Additional file 1.** Interview Guide (English version).

## Data Availability

All data and materials related to the study can be obtained through contacting the first author at ycp232@126.com.

## Abbreviations

HBV: Hepatitis B virus
HIV: Human immunodeficiency virus
MABD: Mutual assistance blood donation
VUBD: Voluntary uncompensated blood donation
WHO: World Health Organization
