# Supplementary material for: Why some donors are more willing to donate platelets? ----a qualitative study on 25 regular platelet donors in Guangzhou, China
Source: BMC Public Health. 2019 Dec 12;19:1671. doi: 10.1186/s12889-019-7783-0 (PMC6909608; doi:10.1186/s12889-019-7783-0)
Supplement: Supplementary file 1 — Additional file 1. Interview Guide (English version). [file 12889_2019_7783_MOESM1_ESM.doc]

**Additional file 1：Interview Guide(English version)**

Inclusion criteria were: 1) >3 times of platelet donation through VUBD and at least once in the last 12 months, and 2) pledged at least one platelet donation within the next year.

1. Demographic information: age, gender, education level, occupation, religious beliefs, family structure, etc.

2. The first experience of donating whole blood(if applicable).

3. The first experience of donating platelets.

4. The first experience of MABD(if applicable).

5. What do you think is the difference between donating whole blood and platelets?

6. What do you think is the difference between MABD and VUBD?

7. What is your family's attitude when you participate in the donation of platelets? What is the attitude of the people around you?

8. Do you know about the blood donation policy? Such as blood donation encouragement policy. Are these attractive to you?

9. Do you have any suggestions to blood donation?

10. Other supplementary questions.
